# FGL1 as a Novel Mediator and Biomarker of Malignant Progression in Clear Cell Renal Cell Carcinoma

**DOI:** 10.3389/fonc.2021.756843

**Published:** 2021-12-09

**Authors:** Zheng Lv, Bo Cui, Xing Huang, Hua-Yi Feng, Tao Wang, Han-Feng Wang, Yun-Dong Xuan, Hong-Zhao Li, Xin Ma, Yan Huang, Xu Zhang

**Affiliations:** ^1^ School of Medicine, Nankai University, Tianjin, China; ^2^ Medical School of Chinese PLA, Beijing, China; ^3^ Department of Urology, The Third Medical Center, Chinese PLA General Hospital, Beijing, China

**Keywords:** epithelial to mesenchymal transition, fibrinogen-like protein 1, progression, clear cell renal cell carcinoma, biomarker

## Abstract

Clear cell renal cell carcinoma (ccRCC), which is the most prevalent renal cell carcinoma subtype, has a poor prognosis. Emerging strategies for enhancing the immune response in ccRCC therapy are currently being investigated. Fibrinogen-like Protein 1(FGL1) is a novel mechanism that tumors may use to evade the immune system by binding LAG-3 and negatively regulating T cells. In this study, we aimed at investigating the underlying mechanism of FGL1 in ccRCC, and its expression and prognostic value. We found that FGL1 was upregulated in tumor tissues and plasma specimens of ccRCC patients. High FGL1 expression predicted a poor prognosis for ccRCC patients. We also discovered that overexpression of FGL1 enhances RCC cell migration, invasion, and metastasis by activating the epithelial-to-mesenchymal transition (EMT). Consistent with these results, we identified a significant positive correlation between expression of FGL1 and EMT-related genes through tissue microarray analysis. Gene-expression analysis revealed that FGL1-deficient ccRCC cell lines had altered transcriptional output in inflammatory response, cell-cell signaling, negative regulation of T cell activation, and intracellular signal transduction. Depletion of FGL1 significantly inhibited tumor growth and lung metastasis in orthotopic xenograft mouse model. Infiltration of myeloid-derived CD11b+ and Ly6G+ immune cells in tumor microenvironment (TME) was strikingly decreased when FGL1 expression reduced. Therefore, increased FGL1 expression in ccRCC is positively correlated with poor prognosis. Mechanistically, FGL1 facilitates the EMT process and modulates TME, which promotes ccRCC progression and metastasis. Consequently, targeting FGL1 can potentially improve clinical outcome of ccRCC patients.

## Introduction

Renal cell carcinoma (RCC) is the sixth most common type of neoplasm in men and the tenth in women worldwide (1). Clear cell renal cell carcinoma (ccRCC) is the dominant histological subtype of RCC, accounting for 70~80% of the cases (2). Approximately 30% of ccRCC patients have metastasis at the time of diagnosis, implying that the capacity of ccRCC to metastasize is high (3). Metastatic RCC (mRCC) has a poor prognosis, with a 5-year survival rate of only 5~10% after diagnosis (4). Besides, conventional chemotherapy and radiotherapy are often ineffective against mRCC (5). In the last decade, the availability of targeted therapies, such as tyrosine kinases inhibitors and mTOR inhibitors has improved the prognosis of mRCC patients (6, 7). However, the initial response rates of targeted therapies in ccRCC is only 10%~30%, and nearly all patients treated with targeted agents eventually experience tumor progression due to acquired resistance (8–10).

Improved understanding of tumor microenvironment has led to the development of immunotherapy in RCC (11). The clinical success of immune checkpoint inhibitors (ICIs) that target programmed death 1 (PD-1)/programmed death ligand 1 (PD-L1) and cytotoxic T-lymphocyte associated protein 4 (CTLA-4) have revolutionized the treatment of ccRCC patients. Immunotherapy-based combinations have become standard of care in patients with advanced RCC, and have shown efficacy and overall survival benefits in the first-line metastatic setting (12, 13). Besides targeting PD-1/PD-L1 pathway, many other molecular mechanisms can help repress immunity in the tumor microenvironment (TME). Wang et al. (14), recently discovered that FGL1, a major LAG3 ligand, is responsible for the inhibitory function of T cells in TME. Therefore, we speculate that FGL1 is also essential in ccRCC.

Epithelial mesenchymal-transition (EMT), the process whereby epithelial cells transform into mesenchymal cells, has been considered to be a key mechanism in tumor invasion and metastasis (15). Cancer cells acquire motile features during EMT when mesenchymal markers such as vimentin, fibronectin, and N-cadherin are upregulated and epithelial markers like E-cadherin are downregulated (16, 17). A recent study showed that FGL1 promotes the progression of gastric cancer by facilitating the EMT process (18), whereas another study had suggested that loss of FGL1 could induce EMT in lung cancer (19). These conflicting results seem to mirror a double-sided role for FGL1 in regulating EMT during cancer progression. However, the specific role and mechanism of FGL1 in the process of EMT remain to be elucidated.

Fibrinogen-like Protein 1 (FGL1), also known as hepatocyte-derived fibrinogen-related protein, is structurally similar to angiopoietin-like proteins. It is mainly secreted by the liver and acts as an autocrine growth factor during liver regeneration by activating the EGFR/ERK cascade (20). It has been discovered that overexpression of FGL1 in gastric cancer is correlated with tumor progression and poor prognosis (18). In addition, the upregulation of FGL1 confers Gefitinib resistance by inhibiting apoptosis in non-small cell lung cancer (21). However, to the best of our knowledge, the expression, biological function, prognostic significance, and molecular mechanism of FGL1 in ccRCC is unclear. In addition, the role of FGL1 in modulating the TME is still largely undefined.

In this study, we determined that FGL1 expression was upregulated in ccRCC resulting in significant correlation with a poor prognosis for ccRCC patients. Besides, FGL1 promoted the migration, invasion, and metastasis of ccRCC cells by facilitating the EMT process. Finally, we revealed that FGL1 stimulated tumor growth *in vivo* by increasing myeloid-derived CD11b+ and Ly6G+ immune cell infiltration in TME. Collectively, these findings provided insights into the functions of FGL1 in tumor progression, suggesting that targeting FGL1 can be a potential therapeutic strategy for ccRCC.

## Materials and Methods

### Patients and Clinical Materials

Tumor specimens were collected from 211 ccRCC patients who underwent surgery at the Urology Department of the Chinese People’s Liberation Army (PLA) General Hospital (Beijing, China) from January 2012 to December 2019. Clinic-pathologic data of ccRCC patients were reported in **Supplementary Table 1**. Cancer tissue samples were pathologically confirmed as ccRCC according to the 2011 Union for International Cancer Control TNM classification of malignant tumors. Forty-three preoperative blood samples of ccRCC patients and 26 blood samples from healthy donors were collected between January and March 2021. The patients signed for consent after being informed about the use of their clinical specimens for scientific research. This study was approved by the ethics committee of the Chinese People’s Liberation Army (PLA) General Hospital.

### Plasmid Constructs

Oligonucleotides targeting human and mouse FGL1 were designed using short hairpin RNA (shRNA) sequences, and synthesized by BGI (Shenzhen, China). Double stranded oligonucleotides targeting FGL1 (shFGL1) and NC shRNA (shNC) were cloned into Plko.1, after annealing. The 1 puro lentiviral vector was digested with EcoRI and BstHI. The sequences of primers used for the shNC, FGL1-sh1, and FGL1-sh2 are listed in **Supplementary Table 2**.

### Cell Culture, Transfection and Infection

Fetal bovine serum (FBS) was purchased from EVERY GREEN (Hangzhou, China). Different types of media including high glucose-DMEM, MEM, RPMI-1640, and McCoy’s 5A were purchased from VIVICUM bioscience (Beijing, China). Similarly, various cell lines including the human embryonic kidney derived cell line HEK293T, human renal tubular epithelial cell line HKC, human ccRCC cell lines SN12, A498, 786O, ACHN, OS-RC-2, Caki-1, and Caki-2 were originally purchased from American Type Culture Collection. All the cells were cultured in medium containing 10% FBS and 1% penicillin/streptomycin in 5% CO_2_ at 37°C. Cell lines HEK293T and SN12 were cultured in high glucose-DMEM medium, A498 and ACHN in MEM medium, 786O and OS-RC-2 in RPMI-1640 medium, and Caki-1 and Caki-2 in McCoy’s 5A medium, respectively.

Transient co-transfection of packing DNA yielded lentivirus in HEK293T cells. Cells were transfected with 6 μg vector plasmids along with 4.5μg psPAX2 and 1.5 μg pMD2-VSVG using the standard calcium chloride transfection method. The calcium transfection kit was purchased from Macgene Biotech (Beijing, China). Viral supernatant containing released viruses was collected at 48h and 72h after transfection, and filtered through a 0.45 μm filter. Target cells were infected at a multiplicity of infection (MOI) of 5 in the presence of 8 μg/mL polybrene for 24h. The infected cells were then selected with 2 μg/mL puromycin (Sigma, USA) for three days. A plasmid overexpressing human FGL1 was purchased from SinoBiological (Beijing, China). Overexpression of FGL1 in SN12 and A498 cells was carried out by transient transfection using Lipofectamine2000.

### MTS Assay

The cells were seeded into 96-well plates (1000 cells/well) and cultured for 12h, 24h, 48h, and 72h. The viability of the cells was assessed using a CellTiter-Blue^®^ (CTB) cell viability assay (CTB169, Promega, Beijing, China). At varying points in time, 20 μl of [3‐(4,5‐dimethylthiazol‐2‐yl) ‐5‐ (3-carboxymethoxyphenyl)-2‐(4-sulfophenyl)-2H-tetrazolium (MTS) reagent was added to each well, and incubated for 2 h at 37°C. The absorbance was measured at a wavelength of 490 nm to detect the OD values. All experiments were performed in triplicate.

### Cell Migration and Invasion Assay

Cell migration and invasion were gauged using transwell migration assay, wound healing assay, and Matrigel invasion assay. For transwell migration assay and Matrigel invasion assay, 5×10^4^ cells suspended in 200 μl of medium without FBS were seeded on the upper chamber (8 μm pore size, 3422, Costar) with Matrigel-uncoated or coated membrane (356224, BD Biosciences). The lower chamber was filled with 500 μl medium containing 20% FBS. Cells were fixed with 4% paraformaldehyde after 16h of incubation at 37°C in 5% CO_2_. The non-migrated cells on the top surface of the membrane were gently removed with a cotton swab, whereas the migrated cells were stained using 0.5% crystal violet (C8470, Solarbio, China), photographed, and counted under a light microscope (200x magnification).

In wound healing assay, cells were seeded in the six-well plate and cultured for 24h at 37°C in 5% CO_2_. A scratch was made using a 200 μl pipette tip when the cells were grown to 90% confluence. The cells were washed with PBS for three times and then incubated in FBS-free medium at 37°C in 5% CO_2_. The gaps between the wound edges were monitored and photographed using an IX2-UCB phase contrast microscope (Olympus, Tokyo, Japan) at 0 and 48 h (200x magnification).

### Western Blot Analysis

Western blot assays were performed using standard techniques as previously reported (22). The information of antibodies was listed in **Supplementary Table 3**. The results of FGL1 protein expression were quantified by the relative gray value, which was calculated as “the gray value of FGL1 protein bands/the gray value of internal control β-tubulin bands”.

### ELISA Analysis

The concentration of FGL1 in the plasma of ccRCC patients and healthy controls was measured using commercially available sandwich ELISA kits (Wuhan Colorful Gene Biological Technology, China).

### Immunohistochemistry (IHC) Staining

Tissue microarray for FGL1 IHC staining was obtained from the tissue bank at Urology Department of the Chinese PLA General Hospital, Beijing, China. The standard protocols were followed as previously described (23). Slides were scanned using Axio Image Z2 Microscope (Zeiss) and TissueFAXS imaging system (TissueGnostics GmbH, Austria). All images were analyzed by TissueQuest and StrataQuest software (TissueGnostics GmbH, Austria). As previously described (24), staining intensity was scored as follows: 0 (negative), 1 (weak), 2 (moderate), and 3 (strong), whereas the staining range was scored as: 0 (0%), 1 (1%–24%), 2 (25%–49%), and 3 (50%–100%). The IHC staining score was obtained by multiplying the intensity scores with staining range. The IHC staining score ranged from 0 to 9. Scores less than two were considered as negative staining, 2–3 indicated weak staining, 4–6 was moderate staining, and >6 was strong staining. Patients with an IHC staining score ≥4 were included in the high expression group, whereas those with an IHC staining score <4 were included in the low expression group.

### Immunofluorescence Staining

Cells from different groups were washed three times with PBS, fixed with 4% paraformaldehyde for 15 min, permeabilized with 0.5% Triton X-100, and then blocked with 5% goat serum for 30 min. Cells were stained with primary antibodies at 37°C for 1h and were incubated with AlexaFluor488 and AlexaFluor594-conjugated secondary antibodies (1:400). Nuclei were counterstained by 0.2 mg/mL DAPI. Samples were imaged with an Axio Image Z2 fluorescence microscope (Zeiss) and analyzed by TissueQuest and StrataQuest software.

### RNA‐Sequence (Seq) Analysis

Total RNA was extracted from cells using TRIzol reagent (Invitrogen). Later, all the samples were sent to BGI Corporation (Shenzhen, China) for further RNA-seq detection and analysis using the MGISEQ-2000 sequencer. The Dr. Tom network platform developed by Beijing Genomic Institute (BGI; http://report.bgi.com) was used to perform the protein–protein interaction (PPI) analysis after defining the differentially expressed genes (DEGs), gene ontology (GO) enrichment analysis, and Kyoto Encyclopedia of Genes and Genomes (KEGG) pathway analysis. The ‘phyper’ function of R software was used for the enrichment analysis, to calculate the P-value and perform FDR correction on the P-value. Q-value ≤ 0.05 was regarded as significant enrichment.

### Quantitative Real-Time PCR (qRT-PCR)

Quantitative real-time PCR was performed on selected genes to verify differential gene expression that was observed through RNA‐seq analysis. Total RNA was extracted with Trizol reagent (Invitrogen). Complementary DNA was synthesized using ProtoScript^®^ II First-Strand cDNA Synthesis Kit (E6300S, NEB, USA). Afterwards, quantitative PCR was performed with NovoStart^®^ SYBR Green Super Mix Plus (E096-01A, novoprotein, China). Relative mRNA expressions were normalized to peptidylprolyl isomerase A (PPIA) with the 2^−ΔΔCT^ method. The used primer sequences were listed in **Supplementary Table 4**. The process was implemented using ABI prism 7500 (Applied Biosystems, USA).

### Orthotopic Xenograft Mouse Model *In Vivo*

The mouse orthotopic xenograft tumor model was prepared using 4~6-week-old BALB/c male nude mice (Vital River Laboratory Animal Technology Co., Beijing, China). For the orthotopic xenograft tumor model, Luc-SN12 shNC or Luc-SN12 shFGL1 cells (1×10^6^) suspended in 100 μl of sterilized PBS + Matrigel (1:1) were injected orthotopically into the subcapsular space of right kidney of nude mice. Surgical procedures were performed under anesthesia by administering an intraperitoneal injection of pentobarbital sodium (80 mg/kg). The mice were maintained at the animal facility of the Cyagen Laboratory, where they were caged and handled under ethical conditions, according to the rules outlined by the International Animal Welfare Recommendations and in accordance with the local institutional animal welfare guidelines. At the end of experiments, the primary and metastatic tumors were harvested, measured, photographed, and fixed for further histopathological analyses.

### Statistical Analyses

Statistical analyses were performed using the GraphPad Prism software version 8.0 (GraphPad software, USA). Normally distributed data were expressed as mean ± standard deviation. Comparisons between two groups were done using unpaired Student’s t-test or Mann–Whitney U test. One-way ANOVA test was used to compare three or more groups. Categorical data was analyzed by the chi-squared test or Fisher’s exact test. Correlations of gene expression were determined with the Pearson’s coefficient test. Kaplan–Meier plots and log-rank tests were used for the overall survival analysis and progression free survival analysis. The univariate and multivariate analyses were executed using the Cox proportional hazards model.

## Results

### FGL1 Is Significantly Upregulated in ccRCC

**Figure 1:**
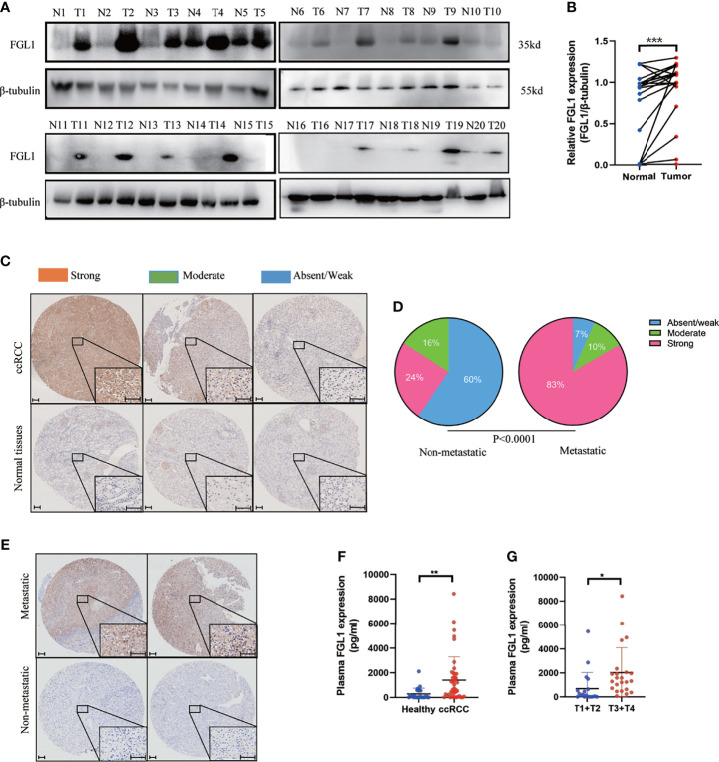
FGL1 is significantly upregulated in ccRCC. **(A, B)** FGL1 protein expression in 20 pairs of ccRCC cancer (T) and adjacent normal tissues (N) by western blot analysis **(A)**. The intensity of bands was quantified using ImageJ software and normalized to β-tubulin **(B)**. The difference between the groups was compared by the student t-test. ***p < 0.001. **(C, D)** Immunohistochemistry (IHC) staining for FGL1 in ccRCC tissues and adjacent normal tissues. FGL1 staining intensity defined into three groups as absent/weak (Blue), moderate (Green), and strong (Red) in clinical ccRCC samples and adjacent normal tissues by IHC **(C)**. Scale bar, 100 μm. Pie chart shows the composition of different staining intensity of FGL1 between metastatic group (n=181) and non-metastatic group (n=30) **(D)**. The difference between the groups was compared by Pearson Chi-square test. **(E)** Distinguished FGL1 protein expression by IHC between representative primary metastatic ccRCC samples (n=2) and nonmetastatic ccRCC samples (n=2). Scale bar, 100 μm. **(F)** Plasma concentrations of FGL1 were measured by ELISA in a cohort of 43 ccRCC patients and 26 healthy donors. The difference between the groups was compared by the student t-test. **p < 0.01, *p < 0.05. **(G)**, Plasma concentrations of FGL1 were measured by ELISA in ccRCC patients of T1+T2 stage (20 cases) vs T3+T4 stage (23 cases). The difference between the groups was compared by the student t-test. *p < 0.05.

Western blotting and IHC were used to examine the expression of FGL1 protein in specimens of ccRCC tumor and adjacent normal tissues. The results showed that FGL1 was significantly higher in cancer tissues than in the adjacent normal tissues (**Figure 1A**). Staining of FGL1 was mainly localized in the extracellular and cytoplasm of ccRCC cells, with varying intensities in different specimens (**Figures 1B–E**). Furthermore, higher FGL1 levels were found to be significantly associated with a strong presence of metastasis (**Figures 1C–E**). In the non-metastatic group, absent/weak immunostaining was observed in 59.7% (108/181) of the tumors, while moderate and strong staining was noted in 40.3% (73/181) of the tumors. By comparison to the non-metastatic group, absent/weak immunostaining was observed in 6.7% (2/30) of the tumors, while moderate and strong staining was noticed in 93.3% (28/30) of the tumors in the metastatic group. This suggested that upregulation of FGL1 may have contributed to the progression of ccRCC by promoting tumor metastasis. In addition, plasma concentration of FGL1 was higher in patients with ccRCC than healthy donors (**Figure 1F**). Patients with high T stage (i.e., T_3_ and T_4_) had higher plasma FGL1 concentration than those with low T stage (i.e., T_1_ and T_2_; **Figure 1G**). These results indicated that FGL1 was significantly upregulated in patients with ccRCC.

### High FGL1 Expression Is Associated With Poor Prognosis in ccRCC Patients

**Figure 2:**
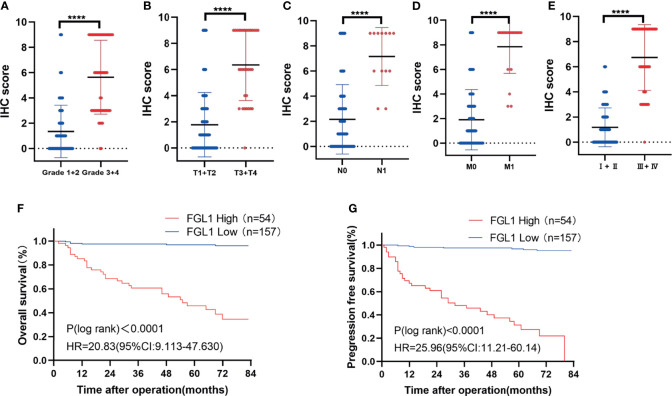
High FGL1 expression is associated with poor prognosis in ccRCC patients. **(A–E)** significantly higher IHC score of FGL1 in patients with high Fuhrman grade (G3 and G4) (n=55) **(A)** advanced T stage (T3 and T4) (n=32) **(B)** positive lymphatic metastasis (N1) (n=13) **(C)** metastasis (M1) (n=20) **(D)** and advanced AJCC stage (III and IV) (n=49) **(E)**. The difference between the groups was compared by the nonparametric test of Mann–Whitney U test. ****p < 0.0001. **(F, G)** Kaplan-Meier curves with log rank test of overall survival **(F)** and progression-free survival **(G)**. High FGL1 expression group (n=54) (red line); low FGL1 expression group (n=157) (blue line).

The correlation between FGL1 expression and the clinicopathologic features of ccRCC patients was shown in **Table 1**. There was significant correlation between FGL1 and age (*P*<0.001), body mass index (*P*<0.001), T stage (*P*<0.001), N stage (*P*<0.001), M stage (*P*<0.001), AJCC stage (*P*<0.001), and Fuhrman grade (*P*<0.001). Immunohistochemistry score of FGL1 was significantly higher in samples from patients with a high T-stage (T3+T4), high Fuhrman grade (G3+G4), N1 stage, M1 stage, and high AJCC stage (III+IV) than in those with a low T-stage (T1+T2), low Fuhrman grade (G3+G4), N0 stage, M0 stage, and low AJCC stage (I+II) (**Figures 2A–E**). The Kaplan–Meier survival analysis indicated that patients with high FGL1 expression had a shorter overall survival and progression-free survival than patients with low FGL1 expression (**Figure 2F, G**). Univariate and multivariate Cox regression analyses indicated that FGL1 expression was an independent prognostic factor for OS (HR = 10.703, P = 0.000) and PFS (HR = 21.954, P = 0.000) in patients with ccRCC (**Table 2**). Therefore, increased FGL1 expression was associated with poor prognosis and might be a novel progression marker for ccRCC.

**Table 1:** Relationship between FGL1 expression and clinicopathological features in patients with ccRCC [n (%)].

Variable	No. of patients (%)			*χ^2^*	*P* value
	Patients	FGL1 High	FGL1 Low		
Age (years)					
≤60	149	22 (14.8)	125 (83.9)	30.563	0.000
> 60	62	32 (51.6)	30 (48.4)		
Gender					
Male	156	43 (66.7)	113 (33.3)	1.222	0.269
Female	55	11 (61.8)	44 (38.2)		
Body mass index					
≤23.9	67	34 (50.7)	33 (49.3)	32.619	0.000
> 23.9	144	20 (13.9)	124 (86.1)		
T stage					
T_1_+T_2_	179	33 (18.4)	146 (81.6)	31.745	0.000
T_3_+T_4_	32	21 (65.6)	11 (34.4)	
N stage					
N0	198	44 (22.2)	154 (77.8)	16.403	0.000
N1	13	10 (76.9)	3 (23.1)		
M stage					
M0	191	37 (19.4)	154 (80.6)	40.948	0.000
M1	20	17 (85.0)	3 (15.0)		
AJCC Stage					
Stage I+II	162	28 (17.3)	64 (88.9)	60.629	0.006
Stage III+IV	49	36 (73.5)	9 (26.5)		
Fuhrman grade					
Grade 1+2	156	20 (12.8)	136 (87.2)	51.266	0.000
Grade 3+4	55	34 (61.8)	21 (38.2)		

**Table 2:** Univariate and multivariable Cox regression models analyzing clinical variables affecting OS and PFS.

	OS				PFS			
Variable	Univariate		Multivariate		Univariate		Multivariate	
	HR (CI95%)	P	HR (CI95%)	P	HR (CI95%)	P	HR (CI95%)	P
Age (years) 1:<60;0:≥60	0.427 (0.226~0.808)	0.009	0.755 (0.389~1.464)	0.405	0.785 (0.424~1.456)	0.443	0.635(0.330~1.224)	0.175
Gender 1:male;0:female	1.279 (0.586~2.791)	0.536	1.601 (0.640~4.006)	0.314	1.283 (0.637~2.587)	0.486	0.669(0.287~1.559)	0.351
BMI 1:<23.9;0:≥23.9	1.732 (0.909~3.299)	0.051	2.088 (0.998~4.371)	0.051	1.731 (0.961~3.118)	0.068	0.412(0.205~0.826)	0.013
T stage 1: ≥T3;0:<T3	4.787 (2.626~8.725)	0.000	0.719 (0.208~2.484)	0.602	4.787 (2.626~8.725)	0.000	1.896(0.581~6.182)	0.289
N stage 1:N1;0: N0	5.960 (2.594~13.695)	0.000	2.343 (0.725~7.577)	0.155	5.474 (2.521~11.888)	0.000	0.663(0.251~1.749)	0.406
M stage 1:M1;0: M0	9.618 (4.833~19.143)	0.000	2.782 (0.727~10.643)	0.135	9.002 (4.467~17.338)	0.000	0.690(0.205~2.323)	0.550
AJCC 1: ≥III;0:<III	8.765 (4.443~17.291)	0.000	0.995 (0.193~5.135)	0.995	10.745 (5.775~20.061)	0.000	0.436(0.103~1.843)	0.259
Fuhrman grade 1: ≤2;0:>2	9.469 (4.685~19.140)	0.000	3.600 (1.646~7.875)	0.001	7.131 (3.900~13.038)	0.000	0.417(0.213~0.817)	0.011
FGL1 1:high;0:low	24.236 (10.013~58.661)	0.000	10.703 (3.876~29.558)	0.000	35.207 (15.273~81.161)	0.000	21.954(8.436~57.134)	0.000

### FGL1 Promote the Migration and Invasion of ccRCC Cells *In Vitro*

**Figure 3:**
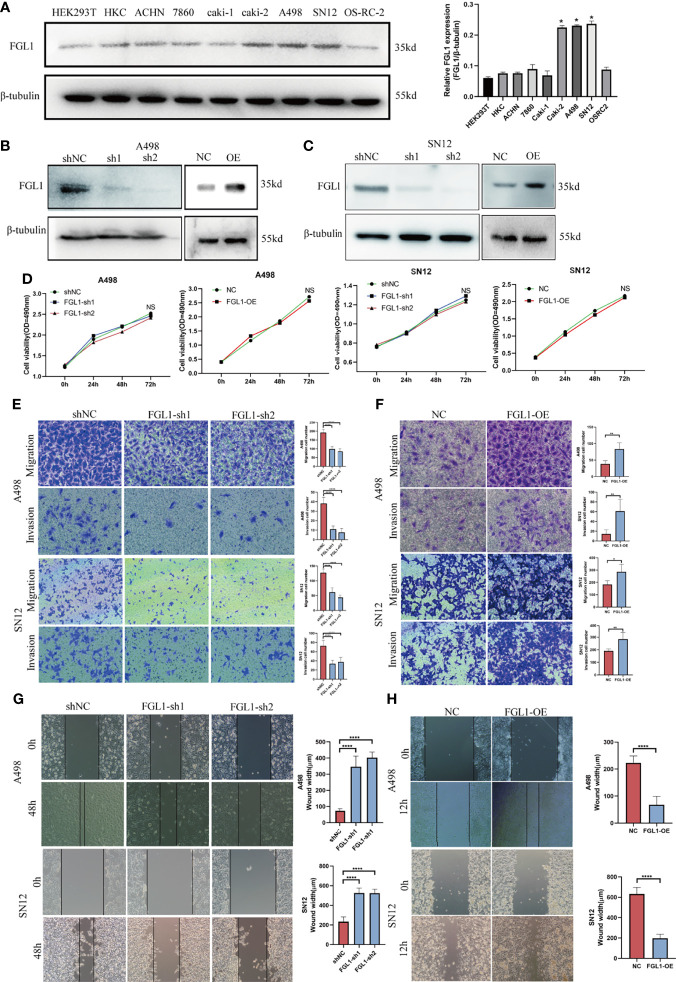
FGL1 promote the migration and invasion of ccRCC cells *in vitro.* **(A)** FGL1 expression was determined by western blot analysis in RCC cell lines (ACHN, 786O, Caki-1, Caki-2, A498, SN12 and OS-RC-2) and normal renal cell lines (HEK293T, HKC) (left). The intensity of bands was quantified using ImageJ software and normalized to β-tubulin (right). *p < 0.05 compared to the HKC cell line. **(B, C)** FGL1 knockdown or overexpression effect was confirmed by Western blot analysis in A498 **(B)** and SN12 cells **(C)**. **(D)** MTS assay to detect the effect of FGL1 knockdown or overexpression on cell proliferation of A498 and SN12 cells at 24h, 48h, and 72h. NS denotes no significant difference between groups. **(E, F)** the effect of FGL1 knockdown or overexpression on migration **(E)** and invasion **(F)** ability of A498 and SN12 cells were examined by Transwell assay. Scale bar, 100 μm. Representative images were taken at 16h after cell migration and invasion. The mean migration or invasion cell numbers of the groups were compared using the student t test. *p < 0.05, **p < 0.01, ****p < 0.0001. **(G, H)** the effect of FGL1 knockdown **(G)** or overexpression **(H)** on migration ability of A498 and SN12 cells were examined by wound healing assay. Scale bar, 100 μm. Wound-healing process measured at 24 hours. The mean wound width of the groups was compared using the student t test. ****p < 0.0001.

In this part, we set out to study the effect of FGL1 on the biological function of ccRCC cells. Firstly, FGL1 was expressed in human normal renal tubular epithelial cell line HK‐2, human embryonic kidney derived cell line HEK293T, and seven ccRCC cell lines (ACHN,786‐O, Caki‐1, Caki‐2, A498, SN12, and OSRC2) at varying degrees, among which A498, SN12, and Caki‐2 cells exhibited the highest FGL1 expression (**Figure 3A**). Then, we validated the knockdown or overexpression effect of FGL1 on ccRCC cell lines (SN12 and A498) by Western blot when transfected with FGL1 shRNA construct or FGL1 overexpression plasmid (**Figure 3B, C**). The MTS assay was introduced to observe the effect of FGL1 on the proliferation of SN12 and A498 cells. The results showed that neither knocking down nor overexpressing FGL1 had significant effect on the proliferation of A498 and SN12 cells at different time points (**Figure 3D**). To investigate the effect of FGL1 on the migration and invasion ability of ccRCC cells, we performed Transwell assay with or without a Matrigel coating. The results showed that when FGL1 was knocked down, the migration rate of A498 and SN12 cells was significantly slower than shNC cells (**Figures 3E, F**). On the other hand, overexpressing FGL1 resulted in a faster rate of migration of A498 and SN12 cells than in shNC cells (**Figures 3G, H**). In the wound healing assay, knocking down FGL1 caused a slower migration of A498 and SN12 cells into the scratched wound than shNC cells (**Figure 3F**), whereas overexpressing FGL1 lead to a faster migration of A498 and SN12 cells migrated into the scratched than of shNC cells (**Figure 3G**). Therefore, FGL1 did not affect ccRCC cell growth/proliferation, but promoted cell migration and cell invasion *in vitro*.

### FGL1 Is Required for EMT Process in ccRCC

**Figure 4:**
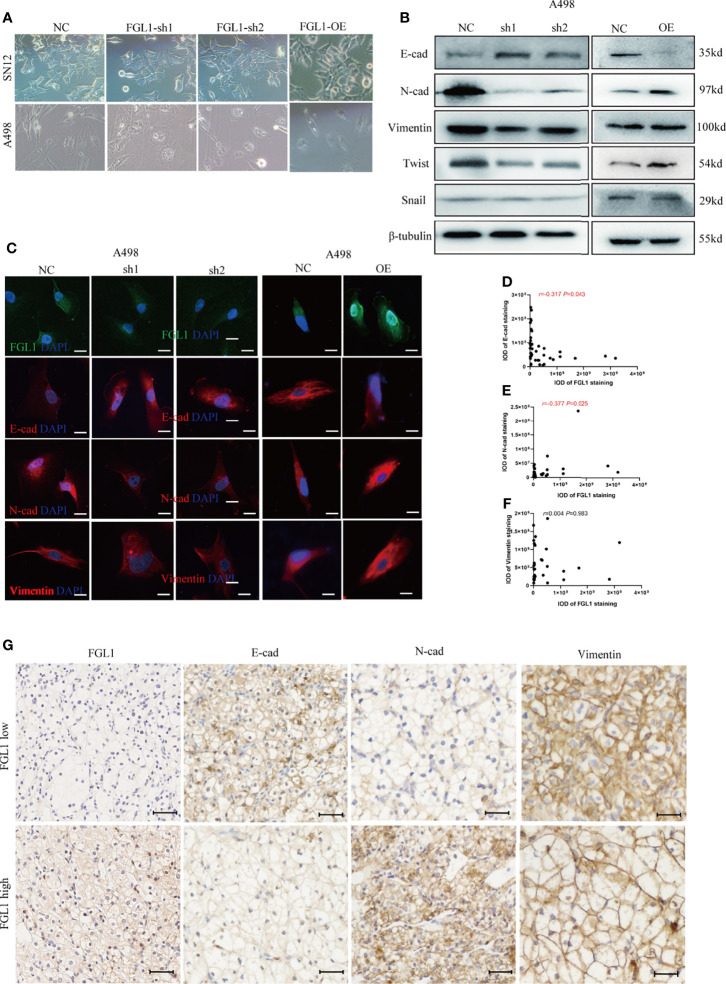
FGL1 is required for EMT process in ccRCC. **(A)** cell morphology of A498 and SN12 cells was observed under bright-field microscopy after FGL1 knockdown or overexpression. **(B)** Western blot analyzed the expression of E-cadherin, N-cadherin, Vimentin, Twist and Snail with FGL1 knockdown or overexpression in A498 cells. **(C)** Immunofluorescence staining of E-cadherin, N-cadherin, Vimentin after FGL1 knockdown or overexpression in A498 cells, with DAPI nuclear staining in blue, FGL1 in green and EMT markers (E-cadherin, N-cadherin, Vimentin) in red. **(D–F)** the correlation between FGL1 and E-cadherin **(D)** N-cadherin **(E)** and vimentin **(F)** expression is represented in a scatter plot. Statistical significance was tested by the Pearson correlation test. In the scatter plot, the symbol r represents the Pearson’s correlation coefficient. IOD, integral optical density. **(G)** Representative images of IHC staining for E-cad, N-cad and Vimentin expression in low- and high-FGL1 tumors. Scale bar, 100μm.

Epithelial mesenchymal-transition (EMT) is a key process for tumor migration, invasion, and metastasis. However, the effects of FGL1 on EMT in ccRCC cells remain unclear. In this study, we observed that after FGL1 knockdown the cells showed a cuboidal cobblestone epithelial shape with tight cell-to-cell adherence, whereas the cells with overexpressed FGL1 had a slender and fibroblast-like shape (**Figure 4A**). These observations suggested that FGL1 participates in the EMT process in ccRCC. As expected, after FGL1 knockdown in A498 and SN12 cells, expression of E-cadherin was upregulated, while it was downregulated in N-cadherin. Moreover, opposite results were observed after overexpression of FGL1 in A498, SN12 cells (**Figure 4B** and **Supplementary Figure S1**). These observations were further validated by immunofluorescence staining (**Figure 4C** and **Supplementary Figure S2**). The features of EMT are regulated by EMT-inducing transcription factors such as Twist and Snail. Our results showed that Twist protein was significantly decreased after FGL1 knockdown in A498 and SN12 cells, and overexpression of FGL1 yielded opposite results. Collectively, these results suggested that FGL1 could promote EMT in ccRCC cells.

To further investigate whether FGL1 could promote EMT in ccRCC, we used IHC to analyze the correlation between FGL1 and EMT markers including E-cadherin, N-cadherin, and Vimentin in ccRCC tissues and paired normal tissues. The results showed that the expression of Vimentin and N-cadherin were significantly higher in ccRCC cancer tissues than in paired normal tissues, whereas the expression of E-cadherin in ccRCC cancer tissues was significantly lower than in paired normal tissues (**Supplementary Figure S3**). Pearson correlation analysis showed that the expression of FGL1 in ccRCC cancer tissues was significantly negatively correlated with E-cadherin expression, whereas significantly positively correlated with N-cadherin expression. No correlation was found between FGL1 and Vimentin expression in ccRCC tissues (**Figures 4D–G**). Altogether, these findings implicate FGL1 with promoting invasion and metastasis by enhancing the EMT process in ccRCC.

### Loss of FGL1 Induces Production of Pro-Inflammatory Cytokines/Chemokines in ccRCC Cells

**Figure 5:**
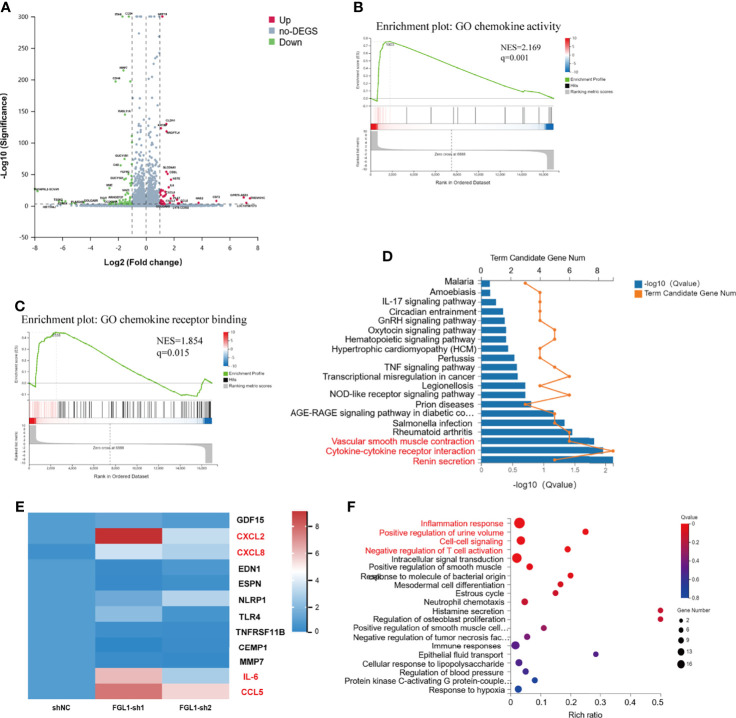
Loss of FGL1 induces production of pro-inflammatory cytokines/chemokines in ccRCC cells. **(A)** Volcano plots were constructed using fold-change values and p-values between shNC and FGL1-sh1 group (log2 FC≥1, FDR< 0.001) by the Dr. Tom network platform. Red represents differentially expressed genes (DEGs) up regulated, blue represents DEG down regulated, and gray represents non-DEG. **(B, C)** GSEA enrichment plot for chemokine activity gene set **(B)** and chemokine receptor binding gene set **(C)** by the Dr. Tom network platform. (Normalized Enrichment Score (NES) > 1, Nominal p-value < 0.05, FDR q-value < 0.25). **(D)** DEGs were analyzed by GO biological process enrichment analysis and showed by histogram chart. The major pathways involved in DEGs were ‘Cytokine-cytokine receptor interaction pathway’, ‘renin secretion’, and ‘vascular smooth muscle contraction’ were showed in red font. **(E)** qRT-PCR validation of the DEGs involved in the cytokine-cytokine receptor interaction pathway. The DEGs, including IL-6, CXCL2, CXCL8, and CCL5, were significantly upregulated in FGL1 knockdown cells and shown in red font. **(F)** Bubble chart for significantly enriched pathway terms using the phyper function in R software to perform the KEGG enrichment analysis. X-axis is the enrichment ratio (Rich Ratio = Term Candidate Gene Num/Term Gene Num), Y-axis is KEGG Pathway.

To determine the transcriptional output regulation after knocking down FGL1 in ccRCC cells, we performed RNA-seq detection in A498 shNC, A498 FGL1-sh1, and A498 FGL1-sh2 cells. Firstly, volcano plots showed that a total of total 119 DEGs including 44 upregulated and 75 downregulated expressed genes were detected in shNC vs FGL1-sh1 group (**Figure 5A**), while a total 97 DEGs including 46 upregulated and 51 downregulated expressed genes were detected in shNC vs FGL1-sh2 group (**Supplementary Figure S4**). Then, Venn diagram showed that 56 genes were shared between shNC vs FGL1-sh1 group and shNC vs FGL1-sh2 group (**Supplementary Figure S5**). We assessed key regulatory pathways using gene-set enrichment analysis (GSEA) and discovered that chemokine activity and chemokine receptor are regulatory targets of FGL1 (**Figures 5B, C**). Moreover, KEGG pathway analysis revealed that the major pathways involved in DEGs were ‘Cytokine-cytokine receptor interaction pathway’, ‘renin secretion’, and ‘vascular smooth muscle contraction’ (**Figure 5D**). The PPI network showed that IL-6 was the most prominent hub with nineteen related proteins, and that CXCL8, CXCL2, and CCL5 were secondary hubs. The majority of these hub genes were found to be involved in the cytokine-cytokine receptor interaction pathway (**Supplementary Figure S6**). Expression of the hub genes that were identified on the RNA-seq was confirmed by qPCR. We found higher levels mRNA for IL-6, CXCL2, CXCL8, and CCL5 in FGL1 knockdown cells than in shNC cells, which was consistent with RNA-seq data (**Figure 5E**).

The DEGs were annotated by GO biological function analysis, cellular component analysis, and biological process analysis. The five most enriched GO terms of the DEGs for the biological process were ‘inflammatory response’, ‘positive regulation of urine volume’, ‘cell-cell signaling’, ‘negative regulation of T cell activation’, and ‘intracellular signal transduction’ (**Figure 5F**). The four most enriched GO terms of the DEGs for cellular component were ‘extracellular space’, ‘basal part of cell’, ‘integral component of plasma membrane’, and ‘extracellular region’ (**Supplementary Figure S7**). The most enriched GO terms of the DEGs for molecular function was ‘cytokine activity’ (**Supplementary Figure S8**).

### FGL1 Silencing Suppressed Tumorigenicity and Metastasis in Orthotopic Xenograft Tumor Model

**Figure 6:**
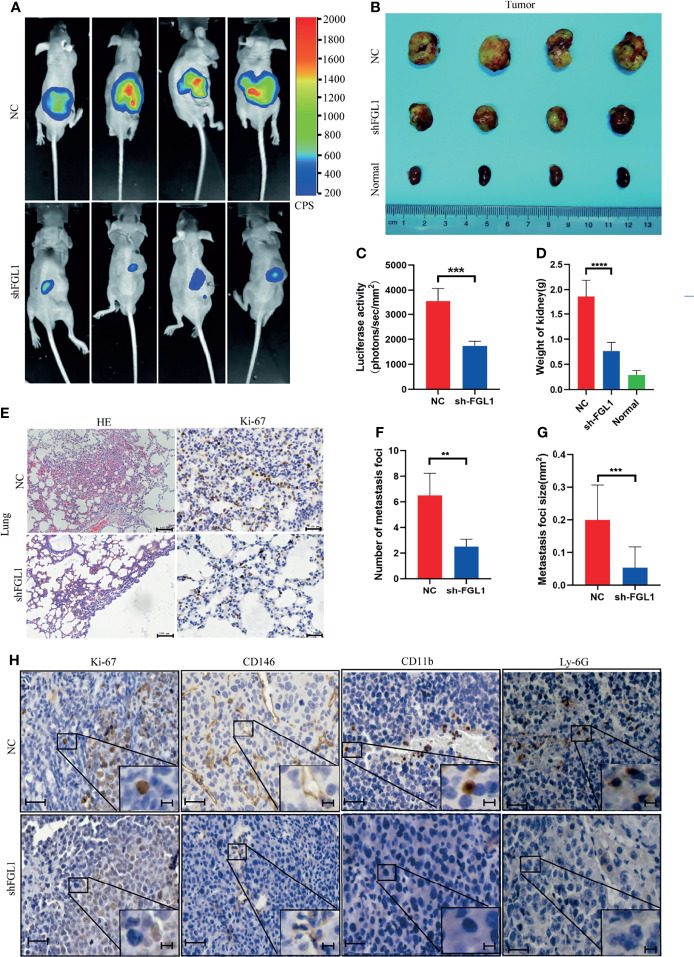
FGL1 silencing suppressed tumorigenicity and metastasis in orthotopic xenograft tumor model. **(A)** Representative bioluminescent images of kidney tumors in shNC (Upper) (n=4) and shFGL1 experimental groups (Lower) (n=4) (four week after cell implantation). CPS, unit of signal intensity in counts per second. **(B)** Gross appearance of kidney tumors in shNC group (n=4), shFGL1 group (n=4) and contralateral normal kidney (n=4). **(C)** Measurement of bioluminescent signals of shNC (n=4) and shFGL1 group (n=4) on day 30. ***P<0.001. **(D)** the histogram shows the weight of tumors in shNC group (n=4) and shFGL1 group (n=4), ***p < 0.001. **(E)** Representative images of hematoxylin-eosin (HE) staining and ki-67 staining of lung metastatic foci in shNC group (n=4) and shFGL1 group (n=4). Scale bar of HE staining, 100 μm. Scale bar of ki-67 staining, 20 μm. **(F, G)** the number **(F)** and size **(G)** of lung metastatic foci was were decreased in shFGL1 group compared to shNC groups. **P<0.01, ***P<0.001. **(H)** Representative images of ki-67 (proliferation marker), CD146 (endothelial cell marker), CD11b (myeloid cell marker) and Ly-6G (neutrophil marker) in tumor tissues from shNC group and shFGL1 group. Scale bar, 20 μm. and scale bar in zoom, 5 μm.

Since FGL1 promotes the migration and invasion of ccRCC cells *in vitro*, we hypothesized that knocking down FGL1 expression will suppress progression and metastasis *in vivo*. We orthotopically injected shFGL1/Luc or shNC/Luc SN12 cells into subcapsular space of nude mice. Four weeks after injection, the bioluminescent signals in the kidneys were significantly higher in the shNC group than in the shFGL1 group (**Figures 6A, C**). In these two groups, all the mice were sacrificed at the end of the fourth week, and the primary tumors are shown in **Figure 6B**. ​The shFGL1 group had lighter and smaller tumors than the control group (**Figures 6D, E**). Our data confirmed that knocking down FGL1 inhibits tumor growth *in vivo*. Gross lung specimens were collected and used to elucidate whether knocking down FGL1 affects metastasis *in vivo* (**Supplementary Figure S9**). We found that mice in the shFGL1 group developed fewer lung metastatic foci than those in the shNC group (**Figures 6E, F**). The average tumor size of metastatic nodules in the shFGL1 group was smaller than in the shNC group (**Figure 6G**). Furthermore, we discovered that the shNC group had more CD146 positive microvessels and a higher positivity rate of CD11b+ and Ly-6G+ cells in tumor tissues than in the shFGL1 group (**Figure 6H** and **Supplementary Figure S10**). Therefore, our findings demonstrated that FGL1 knockdown repressed the tumorigenicity and metastasis properties of ccRCC cells *in vivo*.

## Discussion

Invasion and metastasis, two of the most significant hallmarks of malignant tumors, are major obstacles to the treatment of malignant tumors, and have effects on the prognosis of patients (25). Although surgical resection can cure certain cancers when diagnosed at early stages, metastatic cancer is largely incurable and results in high mortality rate (26). This is likely because the precise mechanisms of metastatic changes during cancer progression are still largely unknown.

Fibrinogen-like protein 1(FGL1), originally named as human hepassocin (HPS), is a liver-secreted protein that plays a critical role in liver regeneration by activating the MAPK pathway to repair damaged hepatocytes (27, 28). Further experiments indicated that FGL1 regulates metabolism by increasing hepatic lipid accumulation and inducing insulin resistance (29). Recent evidence boosted the research of FGL1 in cancer biology by illustrating a new function of FGL1 in immune suppression where it acts as a major inhibitory ligand of LAG-3 and inhibits antigen-specific T cell activation (14). Recent research suggested that FGL1 is upregulated in gastric cancer tissues, while some studies revealed that it is downregulated and possibly acts as a tumor suppressor in hepatocellular carcinoma (18, 30). Accordingly, depending on the cell type and origin of the cancer, FGL1 can be upregulated or downregulated acting as an oncogene or tumor suppressor, thus indicating the complexity and diversity of roles played by FGL1 in different cancers. Currently, the expression and function of FGL1 in ccRCC is poorly understood. Herein, we reported for the first time that FGL1 is upregulated in both cancer tissues and plasma of ccRCC patients. High expression of FGL1 in cancer tissues is closely associated with distal metastasis and fatal outcome in ccRCC patients. Univariate and multivariable Cox proportional hazard regression analyses further showed that, high FGL1 expression in cancer tissues is an independent risk factor for ccRCC patients with poor prognosis, indicating that FGL1 may be an oncogene of ccRCC. Consequently, FGL1 may be used as a new prognostic marker for ccRCC.

Epithelial mesenchymal-transition (EMT) is now widely recognized as an indispensable step in tumor invasion and metastasis (31), and E−cadherin, N−cadherin, and Vimentin are generally accepted as important molecular markers of EMT (32). The loss of E-cadherin, an important feature of EMT, has been linked to invasive and undifferentiated phenotype in malignant tumors (17). Markers such as N-cadherin and vimentin were upregulated during EMT to induce mesenchymal phenotypes and motile behavior (16). Our data showed that overexpression of FGL1 decreased the expression of E-cadherin while increasing the expression of N-cadherin, causing ccRCC cells to evolve into a highly invasive and mesenchymal phenotype. Furthermore, overexpression or knocking down of FGL1 increased or decreased the levels of Twist proteins, respectively. Previous study had indicated that upregulation of Twist proteins resulted in a significant decrease in the expression of E-cadherin and a prominent increase in the expression of N-cadherin, thus promoting the migration and invasion abilities in cancer cells (33). In light of our findings, FGL1 might upregulate Twist to maintain the mesenchymal phenotype and promote the migration and invasion behavior of ccRCC cells. Besides, we also observed a significant association between FGL1 expression and EMT biomarkers in clinical specimens. Therefore, our data elucidated that FGL1 exerts the biological function of promoting tumor cell migration, invasion, and metastasis by facilitating EMT process in ccRCC. However, the detailed mechanism by which FGL1 regulates EMT is unknown, and further research is required.

Numerous studies had reported that FGL1 knockdown either inhibits or promotes tumor cell proliferation. However, our data showed that neither overexpression nor knockdown of FGL1 in ccRCC cell lines had an effect on cell proliferation, but cell migration and invasion were affected. Interestingly, in contrast to the *in vitro* assay, our results showed that FGL1 knockdown inhibited orthotopic tumor growth in xenograft tumor model using T cell-deficient nude mice. We speculated that FGL1 may play an important role in modulating tumor innate immunity. Our results demonstrated that FGL1 knockdown significantly decreased the infiltrated number of myeloid-derived CD11b+ cells and ly6G+ cells in primary tumor tissues. Various types of myeloid cells, such as tumor-associated neutrophils (TANs) and myeloid-derived suppressor cells (MDSC), have been shown to promote tumor progression by inhibiting anti-tumor immunity (34, 35). Neutrophil accumulation in tissue involves different steps including chemotaxis, activation, and transmigration (36, 37). Our study showed that the expression of CD146 was significantly reduced in shFGL1 tumors. Bardin N. et al. (38), reported that CD146, which is found at the junction and apical membrane of human umbilical veins endothelial cells, can contribute to transendothelial migration of monocytes during inflammation. So, knocking down FGL1 can help to repress neutrophil accumulation in tumor by reducing neutrophils transmigration through blood vessels. In addition, our study revealed that some inflammatory cytokines (such as IL-6) and chemokines (such as CXCL2, CXCL8, and CCL5) were elevated after FGL1 knockdown in ccRCC cells. Chemokines, like CXCL2, CXCL8 and CCL5, can mobilize and activate resting NK cells, resulting in tumor cell cytolysis (39). Based on these observations, our study showed that FGL1 achieves its function by influencing the release of cytokines and chemokines from cancer cells, which can dampen anti-tumor immune responses in TME. However, further research in immunocompetent mice is needed to fully understand this effect.

In summary, our report demonstrates that FGL1 is upregulated in ccRCC patients, and that high expression of FGL1 is associated with poor prognosis. Moreover, we validated that FGL1 stimulates the migration, invasion, and metastasis phenotype in ccRCC by promoting the EMT process. Besides mediating T cell suppression, we demonstrated that FGL1 has a novel role in regulating innate immune response. Therefore, targeting FGL1 may help to suppress the progression of ccRCC and improve clinical outcomes. However, many problems remain to be solved. First, the mechanism by which FGL1 regulates the expression of cytokines and chemokines in cancer cells remains largely unknown. Second, future research should focus on how FGL1-regulated cytokines and chemokines modulate immune cell functions. Nevertheless, FGL1 remains a potential therapeutic target in cancer.

## Data Availability Statement

The raw data supporting the conclusions of this article will be made available by the authors, without undue reservation.

## Ethics Statement

Patients were asked to sign an informed consent before inclusion in the study and the study was approved by Ethics Committee of Chinese PLA Hospital. The patients/participants provided their written informed consent to participate in this study. Written informed consent was obtained from the individual(s) for the publication of any potentially identifiable images or data included in this article.

## Author Contributions

The study design, XZ., YH, and ZL. The experiment and performed data analysis, BC and H-YF. The animal experiments: XH, Y-DX, and TW. The clinical data analysis, H-FW and H-YF. The IHC and IF assay: ZL. Manuscript writing, reviewing, and revision: ZL, XM, YH, and XZ. Study supervision: H-ZL, XM, and XZ. All authors contributed to the article and approved the submitted version.

## Funding

This study was financially supported by the National Natural Science Foundation of China (grant number. 81972389 and 81770790).

## Conflict of Interest

The authors declare that the research was conducted in the absence of any commercial or financial relationships that could be construed as a potential conflict of interest.

## Publisher’s Note

All claims expressed in this article are solely those of the authors and do not necessarily represent those of their affiliated organizations, or those of the publisher, the editors and the reviewers. Any product that may be evaluated in this article, or claim that may be made by its manufacturer, is not guaranteed or endorsed by the publisher.

## Abbreviations

FGL1, Fibrinogen-like Protein 1; ccRCC, clear cell renal cell carcinoma; EMT, Epithelial-mesenchymal transition; TME, tumor microenvironment.
