# Supplementary material for: FGL1 as a Novel Mediator and Biomarker of Malignant Progression in Clear Cell Renal Cell Carcinoma
Source: Front Oncol. 2021 Dec 9;11:756843. doi: 10.3389/fonc.2021.756843 (PMC8695555; doi:10.3389/fonc.2021.756843)
Supplement: Supplementary file 1 [file DataSheet_1.pdf]

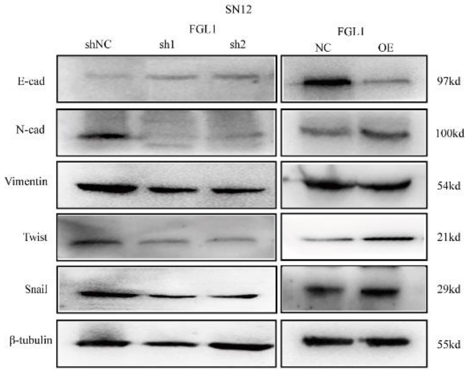

Supplement figure S1, Western blot analyzed the expression of EMT protein markers and EMT-regulating transcription factors with FGL1 knockdown or overexpression in SN12 cells.

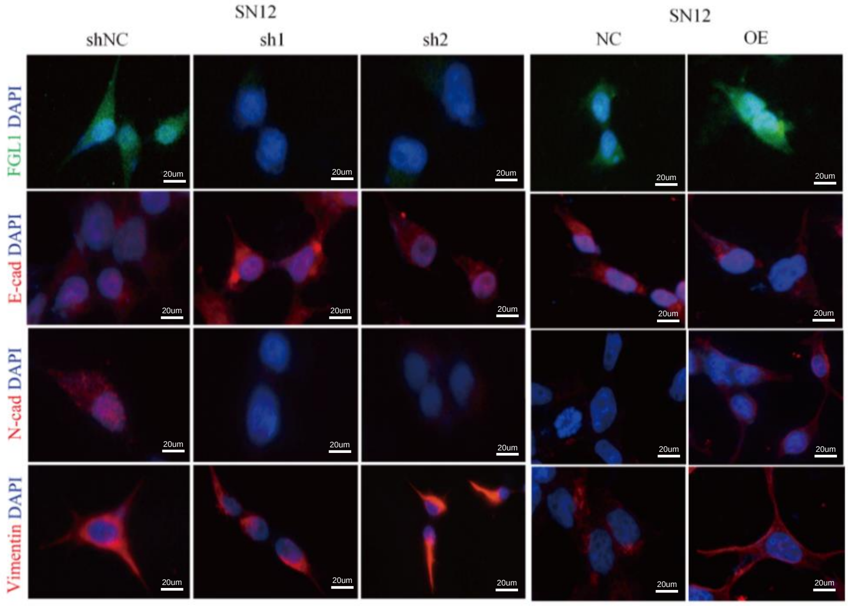

Supplement figure S2, Immunofluorescence staining of EMT protein markers after FGL1 knockdown or overexpression in SN12 cells, with DAPI nuclear staining in blue, FGL1 in green and EMT markers (E-cad, N-cad and Vimentin) in red. Scale bar, 20 μm.

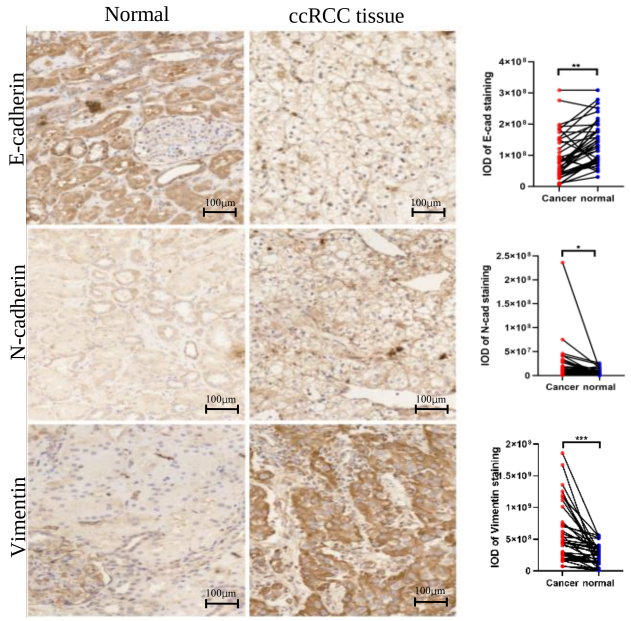

Supplement figure S3, Representative IHC staining of E-cadherin, N-cadherin and Vimentin in ccRCC cancer tissues and paired normal tissues. Scale bar,100μm. Histogram shows the difference of integral optical density (IOD) of E-cadherin (F), N-cadherin (G) and Vimentin (H) between ccRCC tissues and paired normal tissues. ***p < 0.001, **p < 0.01, *p < 0.05.

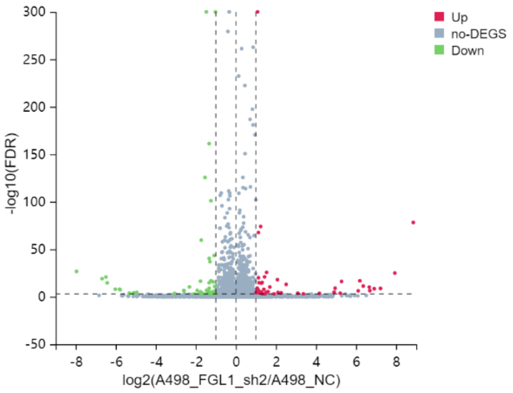

Supplement figure S4, Volcano plots were constructed to examine DEG expression distributions in shNC vs FGL1-sh2 group in A498 cells.

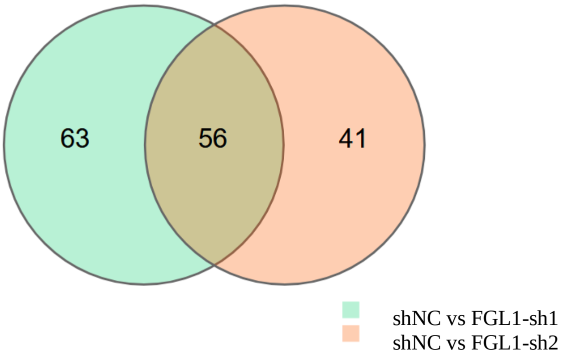

Supplement figure S5, Venn diagram to illustrate the overlapped DEGs between the shNC vs FGL1-sh1 group and shNC vs FGL1-sh2 group (log2 FC≥1, FDR< 0.001).

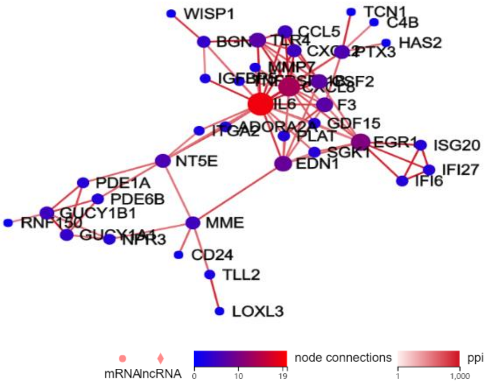

Supplement figure S6, Protein-protein interaction (PPI) network demonstrated potential physical interaction between DEGs.

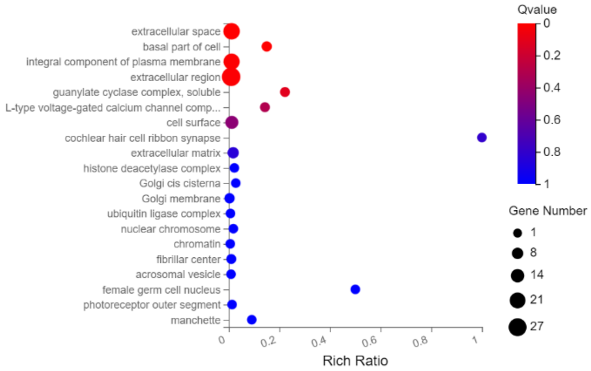

Supplement figure S7, DEGs were analyzed by GO Cellular component Enrichment analysis and showed by bubble chart. X-axis is the enrichment ratio [Rich Ratio = Term Candidate Gene Num/Term Gene Num]. The size of the bubble represents the number of DEGs annotated to a GO Term. The color represents the enriched significance. The redder the color, the smaller the significance value.

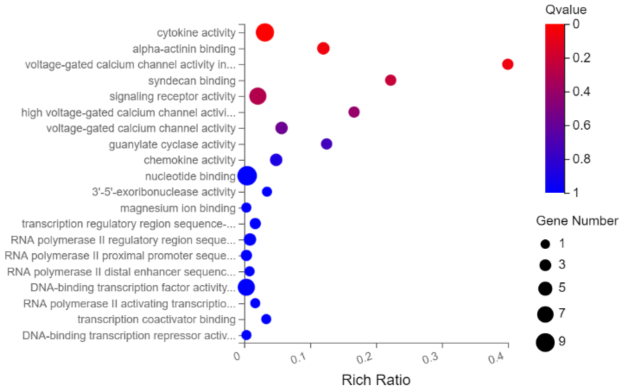

Supplement figure S8, DEGs were analyzed by GO Molecular function Enrichment analysis and showed by bubble chart. X-axis is the enrichment ratio [Rich Ratio = Term Candidate Gene Num/Term Gene Num]. The size of the bubble represents the number of DEGs annotated to a GO Term. The color represents the enriched significance. The redder the color, the smaller the significance value.

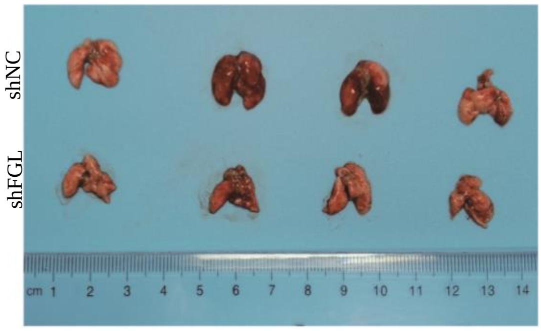

Supplement figure S9, Gross appearance of lungs in shNC group and shFGL1 group.

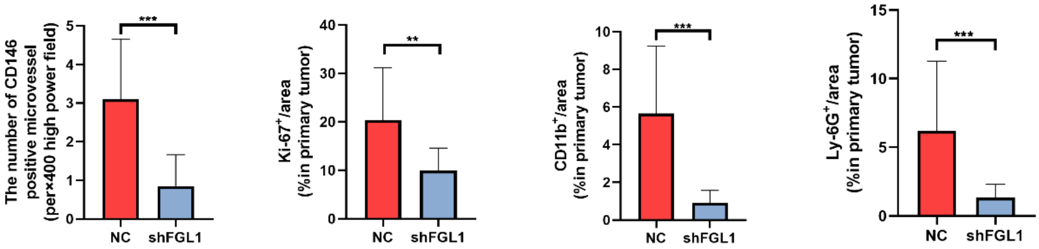

Supplement figure S10, the number of CD146 (endothelial cells marker) positive microvessel, the positive rate of ki-67 (proliferation marker), CD11b (myeloid cell marker) and Ly-6G (neutrophil marker) in tumor tissues were compared between the shNC group and the shFGL1 group. $^{**}p < 0.01$; $^{***}p < 0.001$.
