# Supplementary material for: FGL1 as a Novel Mediator and Biomarker of Malignant Progression in Clear Cell Renal Cell Carcinoma
Source: Front Oncol. 2021 Dec 9;11:756843. doi: 10.3389/fonc.2021.756843 (PMC8695555; doi:10.3389/fonc.2021.756843)
Supplement: Supplementary file 2 [file DataSheet_2.docx]

**Supplementary Table 1. Clinicopathological characteristics of 211 patients of ccRCC**

| **Clinical parameters** | **Variable** | **Total(211)** | **Percentages(%)** |
| --- | --- | --- | --- |
| Age(years) | ≤60 | 149 | 70.6 |
|  | ＞60 | 62 | 29.4 |
| Gender | Male | 156 | 73.9 |
|  | Female | 55 | 26.1 |
| Body mass index | ≤23.9 | 67 | 31.8 |
|  | ＞23.9 | 144 | 68.2 |
| T stage | T1+T2 | 179 | 84.8 |
|  | T3+T4 | 32 | 15.2 |
| N stage | N0 | 198 | 93.8 |
|  | N1 | 13 | 6.2 |
| M stage | M0 | 191 | 90.5 |
|  | M1 | 20 | 9.5 |
| AJCC stage | Stage Ⅰ+Ⅱ | 162 | 76.8 |
|  | Stage Ⅲ+Ⅳ | 49 | 23.2 |
| Fuhrman grade | Grade 1+2 | 156 | 73.9 |
|  | Grade 3+4 | 55 | 26.1 |
| FGL1 | High | 54 | 25.6 |
|  | Low | 157 | 74.4 |

**Supplementary Table 2** **shRNA primers**

| **Primer** | **Sequence(5’-3’)** | |
| --- | --- | --- |
| shNC F | | CCGGGTGGACTCTTGAAAGTACTATCTCGA  GATAGTACTTTCAAGACTCCACTTTTTG |
| shNC R | | AATTAAAAAGTGGACTCTTGAAAGTACTATCT  CGAGATAGTACTTTCAAGAGTCCAC |
| FGL1-sh1 F | | CCGGGAAGTCCAGTTCCTTGATAAACTCGAG  TTTATCAAGGAACTGGACTTCTTTTTG |
| FGL1-sh1 R | | AATTCAAAAAGAAGTCCAGTTCCTTGATAAACTCGA  GTTTATCAAGGAACTGGACTTC |
| FGL1-sh2 F | | CCGGGACAGAGATCATGACAACTATCTCGAGATAGTT  GTCATGATCTCTGTCTTTTTG |
| FGL1-sh2 R | | AATTCAAAAAGACAGAGATCATGACAACTATCTCGAG  ATAGTTGTCATGATCTCTGTC |
| Mouse FGL1-sh1 F | | CCGGGTATGCAGATTGTTCAGAGATCTCGAGATCTCT  GAACAATCTGCATACTTTTTG |
| Mouse FGL1-sh1 R | | AATTCAAAAAGTATGCAGATTGTTCAGAGATCTCGAGA  TCTCTGAACAATCTGCATAC |
| Mouse FGL1-sh2 F | | CCGGCCATTGCTCTGATGATGGGAACTCGAGTTCCCATC  ATCAGAGCAATGGTTTTTG |
| Mouse FGL1-sh2 R | | AATTCAAAAACCATTGCTCTGATGATGGGAACTCGAGT  TCCCATCATCAGAGCAATGG |

**Supplementary Table 3. Antibodies**

| Name | Manufacturer | Number | Type | Usage |
| --- | --- | --- | --- | --- |
| FGL1 | Santa Cruz | sc-514057 | Monoclonal | IF,WB,IHC |
| E-cadherin | Proteintech | 20874-1-AP | Polyclonal | IF,WB,IHC |
| N-cadherin | Proteintech | 22018-1-AP | Polyclonal | IF,WB,IHC |
| Vimentin | Proteintech | 10366-1-AP | Polyclonal | IF,WB,IHC |
| Snail | CST | 3879 | Monoclonal | WB |
| Twist | CST | 69366 | Monoclonal | WB |
| β-tubulin | Easybio | BE0025-10/100 | Monoclonal | WB |
| CD11b | Abcam | ab133357 | Monoclonal | IHC |
| Ly6G | Abcam | ab238132 | Monoclonal | IHC |
| Ki-67 | CST | 12075 | Monoclonal | WB |
| AlexaFluor488  anti-Mouse | Invitrogen | A32723 | Monoclonal | IF |
| AlexaFluor594 | Invitrogen | A32754 | Monoclonal | IF |

WB: Western blot; IHC: Immunohistochemistry; IF: Immunofluorescence; shRNA: short hairpin RNA.

**Supplementary Table 4. qRT-PCR primers**

| **Name** | **Primer sequences (5’-3’)** |
| --- | --- |
| FGL1 | F: ATGGCAAAGGTGTTCAGTTTCA R: ACAATCTGCATACTGCCTCTTG |
| PPIA | F: CCCACCGTGTTCTTCGACATT R: GGACCCGTATGCTTTAGGATGA |
| GDF15 | F: GACCCTCAGAGTTGCACTCC R: GCCTGGTTAGCAGGTCCTC |
| CSF2 | F: TCCTGAACCTGAGTAGAGACAC R: TGCTGCTTGTAGTGGCTGG |
| CXCL2 | F: TCAATGTGACGGCAGGGAAAT R: ACACAGAGGGAAACACTGCAT |
| CXCL8 | F: TTTTGCCAAGGAGTGCTAAAGA R: AACCCTCTGCACCCAGTTTTC |
| EDN1 | F: AGAGTGTGTCTACTTCTGCCA R: CTTCCAAGTCCATACGGAACAA |
| ESPN1 | F: CAGAGTGCAGGACAAAGACAA R: GCAGCGTAGTGGATAGGCAG |
| NLRP1 | F: GCAGTGCTAATGCCCTGGAT R: GAGCTTGGTAGAGGAGTGAGG |
| TLR4 | F: AGACCTGTCCCTGAACCCTAT R: CGATGGACTTCTAAACCAGCCA |
| TNFRSF11B | F: GCGCTCGTGTTTCTGGACA R: AGTATAGACACTCGTCACTGGTG |
| CEMP1 | F: CCTGGGGATGTGTGAAGAGAT R: GGTTTGCTTTCTGCTCCTGAG |
| MMP7 | F: GAGTGAGCTACAGTGGGAACA R: CTATGACGCGGGAGTTTAACAT |
| IL-6 | F: ACTCACCTCTTCAGAACGAATTG R: CCATCTTTGGAAGGTTCAGGTTG |
| CCL5 | F: CCAGCAGTCGTCTTTGTCAC R: CTCTGGGTTGGCACACACTT |
